# Factors Predisposing Out-of-School Youths to HIV/AIDS-related Risky Sexual Behaviour in Northwest Ethiopia

**Published:** 2007-09

**Authors:** Hibret Alemu, Damen Haile Mariam, Kassahun Abate Belay, Gail Davey

**Affiliations:** 1 Department of Community Health, Addis Ababa University, PO Box 11950, Addis Ababa, Ethiopia; 2 USAID-Ethiopia, PO Box 1014, Addis Ababa, Ethiopia

**Keywords:** Acquired immunodeficiency syndrome, Cross-sectional studies, Descriptive studies, HIV, Risk factors, Sex behaviour, Sexually transmitted diseases, Ethiopia

## Abstract

Ethiopia is a developing country with a demographic profile dominated by a young population. Due to biological, psychological, sociocultural and economic factors, young people, particularly those aged 15–24 years, are generally at a high risk of HIV/AIDS and other reproductive health problems. This paper presents results of a cross-sectional descriptive study conducted in Bahir Dar town, northwest Ethiopia, to assess factors that predispose out-of-school youths to HIV/AIDS-related risk behaviours. Both quantitative and qualitative data-collection methods were employed to conduct the study. For quantitative data collection, a household interview survey was conducted among 628 out-of-school youths, aged 15–24 years, within the 17 *kebeles* (villages) of the town. The number of respondents in each *kebeles* was assigned proportional to the size of *kebeles*, and the required numbers of respondents within each *kebeles* were selected through a systematic random-sampling technique. Qualitative data were collected by conducting five focus-group discussions with 46 participants and in-depth interviews with 10 participants. Institutional ethical clearance and informed verbal consent from the study participants were obtained before undertaking the study. Of the 628 study subjects, 64.8% had experienced sexual intercourse at the time of the survey. The mean age at first sexual commencement was 17.7 (+2) years. Of those sexually active, 33% had sexual intercourse with non-regular partners (the proportions were 40.6% among males and 24.7% among females, suggesting that males tended to be about two times more likely to have sex with non-regular sexual partners than females (odds ratio=1.78, with 95% confidence interval 1.16−2.73). Furthermore, consistent condom-use among those who had sex in exchange for money was low (36%). Alcohol intake, chewing of khat (a green leaf), low educational background, and being male were significantly associated with having sex with either a commercial or a non-regular sexual partner. In view of the magnitude of high-risk sexual behaviours among out-of-school youths that may expose them to HIV/AIDS and other sexually transmitted infections, efforts need to be exerted to deal with the identified predisposing factors and to address the problems of idleness, lack of jobs, and hopelessness.

## INTRODUCTION

The youth constitutes the population aged 15–24 years. As a sociocultural phenomenon, this period is characterized as a stage in which one is confronted with role-models for emulating life in adulthood and with the major symbols and values of one's culture and community (1).

Worldwide, there are more than one billion people within the ages of 15–24 years, most of whom live in developing countries. Young people constitute one-third of the total population in Ethiopia. This number is expected to grow from 20.3 million in 2000 to 25 million in 2010 (2). It is a transition period in life from dependent childhood to self-reliant adulthood for joining the labour force. It is at this period that young persons achieve the highest stage of cognitive and physical development and strive to define their self-identity. A need for independence is also one of the features of this age-group, which sometimes may be associated with behaviours of rebelliousness, running away from home, giving up schooling, and even getting married without necessary preparation and parental consent (1).

In general, young people are at a high risk of HIV/AIDS and other reproductive health problems. They generally exhibit behaviours and personality patterns due to biological, psychological, sociocultural and economic factors that are peculiar to their age-cohort. Lack of feeling of mutuality in heterosexual relationship, the tendency for using young girls for immediate sexual gratification, and superficial closeness in the context of randomized sexual relationship are the problems reported by young males (1). Studies conducted in different parts of Africa, including Ethiopia, have shown that most youths indulge in risky sexual behaviour at an early age, often with little regard to the possible consequences (3). Sexual commencement at an early age with limited insight as to the consequences and the low rate of consistent condom-use are among the factors putting youths at a risk of HIV/AIDS. This situation is also further aggravated by the overall socioeconomic environment and the prevalence of harmful traditional practices (4-13).

Ethiopia is among the least-developed countries with multi-faceted reproductive health problems, especially among the youth. Childbearing begins early in life, about 45% of total births in the country occurring among adolescent girls and young women. Commercial sex prevails in urban areas, and sexual violence against women and young girls is a common phenomenon. The seroprevalence of HIV was estimated at 3.5% in 2005. The situation is aggravated by the overall poor socioeconomic environment and harmful traditional practices (6). Thus, identifying the specific factors that predispose youths to risky sexual behaviours will make an important contribution to current efforts in the prevention and control of HIV/AIDS, sexually transmitted infections, and other reproductive health problems.

Bahir Dar town, the study area, is among those with the highest urban HIV prevalence in Ethiopia. The prevalence of HIV was highest in 1992–1993 (13.0%) and has also increased to 20.8% in 1999–2000 (even higher than the rate in Addis Ababa. In particular, out-of-school youths had a 13-time higher chance of being infected with HIV than youths attending school (14). These were the findings that influenced the present study to focus on the assessment of predisposing risky behaviours in a town highly affected by HIV.

The main objective of this study was to identify the factors predisposing out-of-school youths to HIV/AIDS-related risky sexual behaviours in a major urban centre in northwest Ethiopia.

## MATERIALS AND METHODS

The study was a descriptive cross-sectional survey using both quantitative and qualitative methods for data collection. It was conducted during January-March 2003 in Bahir Dar, a regional town in northwest Ethiopia that had the highest reported HIV prevalence rate (23.4%) among the national antenatal care-based sentinel surveillance sites in 2002 (15). At the time of the study, the total population of the town was 153,294 (72,446 males and 80,848 females), of which 40,572 (17,775 males and 22,797 females) were aged 15–24 years.

The study population included all out-of-school youths residing in the town, the inclusion criteria being: age 15–24 years and not attending school or any vocational training at the time of the study. The sample population constituted 628 youths selected from all the 17 kebeles (villages) of the town proportional to the size of the population size of each kebele. Within each kebele, the required numbers of respondents were randomly selected. The first household within a ‘kebele’ was selected by rolling a stick standing at the centre of that kebele and following the random direction of the stick. Once the first household is selected, the consecutive households were systematically picked by adding ‘n’ to the one previously selected (‘n’ being the number of households in the kebele divided by the required number of households from that particular kebele). If there were more than one out-of-school youths in a household, one of them was selected randomly using a lottery method.

For the qualitative part of the study, five focus-group discussions were held with 46 participants (24 males and 22 females), and 10 in-depth interviews with six female and four male out-of-school youths were conducted. Each focus-group discussion session took about one to one and half hours.

Sexual behaviour (sexual commencement at early age), sexual practices (peno-vaginal or peno-anal, and oral), type and number of sexual partners (whether it is with non-regular and multiple partners), consistent condom-use, and history of sexually transmitted diseases (STDs) were the major outcome variables. In addition, education level, sociodemographic characteristics, history of substance abuse, and service-seeking behaviour were the major independent variables identified.

Collected data were entered into statistical programmes (Epi Info 2002 and SPSS version 10) for conducting appropriate descriptive, bivariate (odds ratios) and multivariate (multiple logistic regression) analyses.

Ethical clearance for the study was obtained from the Ethical Review Committee of the Faculty of Medicine at Addis Ababa University. Full information was given to the study participants on the purpose and nature of the research before asking their consent. Enrollment was voluntary, and participants were told of their right not to participate in the study if they do not want to. Confidentiality of the information obtained was also strictly maintained.

## RESULTS

### Results of the quantitative survey

The total response rate was 96.76%. Of the 628 study participants, 324 (51.6%) were males and 304 (48.4%) were females. Two hundred thirty-one (36.8%) of the respondents were aged 15–19 years, while 396 (63.1%) were aged 20–24 years. Their mean age was 20.31+0.09 years (Table 1).

**Table 1:** Sociodemographic characteristics of study participants (out-of-school youths), Bahir Dar, 2003

Variable	Number	Percentage
Sex		
Male	324	51.6
Female	304	48.4
Total	628	100
Age-group (years)		
15–19	231	36.8
20–24	396	63.1
Total	628	100
Religion		
Orthodox	498	79.3
Muslim	83	13.2
Protestant	25	4
No religion	11	1.8
Catholic	7	1.1
Total	628	100
Educational status		
Did not attend formal education	17	4.3
Completed up to junior school (1 to 6 grade)	113	18
Completed up to secondary school (7 to 8 grade)	329	52.4
Completed above senior secondary school (>9^th^)	159	25.3
Total	628	100
Marital status		
Married	142	22.6
Not married	486	77.4
Total	628	100

Of the 628 respondents, 403 (64.2%) had already experienced sexual intercourse at least once. The mean age at first sexual commencement was 17.7 (±2) years. Of the 277 unmarried males, 166 (59.9%) and of the 204 unmarried females, 97 (47.5%) had ever had sexual intercourse. Of the 486 unmarried out-of-school youths, 241 (49.6%) had sexual intercourse at least once in the 12 months preceding the survey. Furthermore, 130 (40.6%) of the 320 males and 74 (24.7%) of the 300 females (who responded to this specific questionnaire item) had intercourse with non-regular partners, suggesting that males tended to be about two times more likely to have sex with non-regular sexual partners than females (odds ratio [OR]=1.78, 95% confidence interval [CI] 1.16–2.73). In addition, a statistically significant association was also observed between education level of youths and their sexual behaviour. Out-of-school youths educated below 6th grade were more than two times at risk of having sex with a non-regular partner and for exchange of money than youths who were educated above 9th grade (OR=2.35, 95% CI 1.29–4.29) (Table 2).

**Table 2:** Multiple logistic regression of sociodemographic variables with risky sexual behaviour (out-of-school youths), Bahir Dar, 2003

Have sex with non-regular sexual partner within the previous 12 months							
Variable	Yes (%)	No (%)	Total	Crude OR (95% CI)		Adjusted OR (95% CI)	
Sex							
Female	74 (24.3)	226 (75.7)	304	1		1	
Male	130 (40.1)	190 (59.9)	320	2.08	(1.47–2.93)	1.78	(1.16–2.73)
Age (years)							
15–19	61 (26.4)	170 (73.6)	231	1		1	
20–24	143 (36)	254 (64)	397	1.57	(1.10–2.25)	1.35	(0.87–2.09)
Education							
>9^th^ grade	41 (25.9)	117 (74.1)	158	1		1	
7^th^-8^th^ grade	105 (31.9)	224 (68.1)	329	1.96	(1.2–3.19)	1.26	(0.76–2.10)
<6^th^ grade	57 (40.7)	84 (59.3)	141	1.33	(0.87–2.04)	2.35	(1.29–4.29)
Chew *khat∗*							
No	61 (15.9)	322 (84.1)	239	1		1	
Yes	143 (59.8)	96 (40.2)	383	7.86	(5.39–11.45)	4.98	(3.27–7.58)
Drink† alcohol							
No	98 (21.5)	358 (78.5)	456	1		1	
Yes	104 (65)	56 (35)	160	5.60	(3.88–8.06)	2.78	(1.83–4.23)

∗ As some youths did not want to respond to some issues, totals may vary

† As some youths did not want to respond to some issues, totals may vary

CI=Confidence interval; OR=Odds ratio

Of the 356 study participants reporting sexual partners, 263 (73.9%) had only one, 43 (12.1%) had two, and the remaining 50 (14%) had more than two sexual partners. About 59% of those who reported having a regular sexual partner claimed to openly discuss sexuality issues with their partners. Those who did not report to openly discuss sexuality issues with their partners were also more than four times likely to have multiple sexual partners than those who claimed to openly discuss sexuality issues with their partners (OR=4.7, 95% CI 2.82–7.80).

Of those who had sex within the 12 months preceding the survey, 112 (40 females and 72 males) also had sex in exchange for money (males with commercial sex workers). Of these 112 participants, 41 (37%) used a condom every time, 26 (23%) had never used a condom, and the remaining 35 (31%) used a condom sometimes.

Two hundred sixty-four (42%) of the 624 study participants reported that they never drank any kind of alcoholic drinks, 113 (18%) drank alcohol about once a week, 85 (14%) drank twice a week, and the remaining 75 (12%) drank alcohol daily. Males were twice as likely to drink alcohol at least once a week than females (OR=1.98, 95% CI 1.423–2.756). Furthermore, a statistically significant association was observed between intake of alcohol and sexual behaviour of youths. Those out-of-school youths who drank alcohol at least once a week were more than three times likely to report encountering a discharging ulcer on their genital tract (OR=3.93, 95% CI 2.21–6.99) and about three times more likely to have sex either with non-regular partners or in exchange for money (OR=2.78, 95% CI 1.83–4.23) than those who did not report consumption of alcohol.

When we assessed the prevalence of khat-chewing [(Catha edulis) a green leaf that is habitually chewed among people in Ethiopia, Kenya, Somalia, and Yemen. The active ingredient in khat that has a stimulant effect is nor-pseudoephedrine], 239 (38%) of the out-of-school youths chewed khat at least once within the 12 months preceding the survey. One hundred fifty-one (63.2%) of these were males, and 88 (36.8%) were females (OR=2.15, 95% CI 1.54–2.99). In addition, 39.5% of those out-of-school youths who chewed khat also reported that it increased their sexual desire. Adjusting for possible confounding sociodemographic variables, it was found that those who chewed khat were about six times more likely to have had sex either with non-regular partners, including commercial sex workers than those who did not report chewing khat (Table 3). Furthermore, a significant association was observed between frequencies of khat-chewing and intake of alcohol.

**Table 3:** Regression analysis of *khat*-chewing and alcohol consumption with risky sexual behaviour (out-of-school youths), Bahir Dar, 2003

Risky sexual behaviour	Chew *khat* vs do not chew		Drink alcohol vs do not drink	
	OR	95% CI	OR	95% CI
Ever had sexual intercourse	5.43	3.35–8.80	3.3	1.82–5.99
Exchange sex for money	5.99	3.5–10.26	2.29	1.39–3.72
Had sex with non-regular partner	6.28	2.9–13.5	2.77	1.47–5.22
Encountered ulcer on the genitalia	3.75	2.43–5.79	3.91	2.50–6.12

CI=Confdence interval; OR=Odds ratio

Five hundred ninety-nine (96%) of the 622 study participants who responded to this specific question had ever heard about sexually transmitted diseases, while 60 (9.6%) of them also reported a history of discharging genital ulcer at least once in the 12 months preceding the survey. Of these 60 participants, 33 (55%) were males, and 27 (45%) were females. The khat-chewers were also about four times more likely than non-chewers to report a history of discharging genital ulcer during the 12 months preceding the survey (Table 3).

### Results of focus-group discussions and in-depth interviews

All participants of the focus-group discussions and in-depth interviews agreed that the pattern of the HIV/AIDS epidemic was getting worse over time, as they are observing the disease seriously affecting the young and productive population groups in the town. The in-depth interview participants further mentioned the minimum age at first sexual commencement as being 14 years and the fact that sexual intercourse is usually started because of peer pressure. In addition, most in-depth interview respondents did not use a condom during their first sexual encounter and during their current sexual practices. The reasons for not using condoms were: it is not comfortable, it decreases sexual satisfaction, and they do not think it is necessary during sexual act with regular (non-spousal) sexual partners.

All focus-group discussion and in-depth interview participants agreed that the key predisposing risk factor putting out-of-school youths at an increased risk of HIV/AIDS in the study town is the increasing number of places for consuming alcohol and khat. It was also reported that there was a trend among youths for taking too much alcohol after chewing khat to avoid the problem of sleep disturbance and anxiety that usually follows the consumption of khat. Taking too much alcohol, in turn, was reported to result in lack of inhibition from indulging in unprotected sex. There was no difference of response by age or gender of the participants.

## DISCUSSION

According to the findings of this study, a substantial proportion of out-of-school youths, especially males, were involved in unsafe sexual intercourse with non-regular partners. Moreover, the habit of abusing substances, such as alcohol and khat, seemed to have contributed to risky sexual behaviour by out-of-school youths in the study area. This finding is comparable with results of other studies in Ethiopia (7–12).

The cultivation and consumption of the stimulant leaf khat (Catha edulis Forsk) is widespread in several countries of East Africa and the Arabian Peninsula, including Ethiopia. Traditionally, it is commonly used for prayer and during Moslem fasting seasons. Although khat in Ethiopia is a cash crop and an important source of foreign exchange next to coffee and skin and hides, it is considered an illicit drug as it contains cathinone, an active brain stimulant that is similar in structure and pharmacological activity to amphetamine (7,8,13,16). As a result, insomnia is a common problem after the use of khat, and users usually tend to consume alcohol afterwards. Khat-chewing in some areas occurs with the use of other substances, such as cigarettes and cannabis (17).

Alcohol makes it difficult for the dependent user to judge what is right or wrong, what is good or bad, and what is moral or immoral. Therefore, it undermines judgment and reduces people's ability not to indulge in unsafe and risky sexual practices that facilitate the transmission and spread of HIV/AIDS (18). Other studies have also reported that substance abuse increases the sexual desire of users and that condoms often do not get used when people are drinking or using drugs with resulting weakening of ego controls and eliciting behaviours likely to increase the probability of exposure to HIV (19,20).

In an earlier study in Addis Ababa, Ethiopia, frequent drinking of alcohol and use of drugs, including khat-chewing, were positively and significantly associated with having sex with a commercial sex partner and with HIV seropositivity (21). In Tanzania, Kenya, and the USA, the frequent uses of alcohol, tobacco, and drugs were the most important predictors of multipartner sexual activity among adolescents and youths (22).

In conclusion, the findings of this study have shown that out-of-school youths in the study area practise multipartner sexual activity without consistent condom-use and, therefore, are at a risk of HIV/AIDS and other sexually transmitted infections. In addition, many out-of-school youths reported the habit of substance abuse in terms of drinking alcohol and chewing khat which were significantly associated with risky sexual practices. Although the study did not address the basic causes of such behaviours (substance abuse) by the out-of-school youths, these may be, among others, the feeling of hopelessness due to lack of jobs and adequate recreational facilities. Therefore, there is a need to further explore the basic causes that lead the out-of-school youths to such high-risk behaviours, so that every effort can be made to address these underlying issues by the community, government and non-government agencies in the area.
